# 

**Published:** 1909-05-22

**Authors:** 


### Bequests and Donations.

Lieut.-Col. James Miller, of Wheatley, Oxon., and Portland Place, W., formerly of the 11th Hussars, left £1,000 to the Evans Cottage Homes, Birmingham, and £500 to the Radcliffe Infirmary, Oxford. Mr. Robert Low, of Palace Court, Bayswater, W., left £1,000 to the Stationers' Company, upon trust to apply the income annually at its discretion in charitable gifts; also £500 each to the Westminster Hospital and the Brompton Hospital for Consumption; and £200 each to St. Mary's Hospital, Paddington, and the West London Hospital. Mr. Benjamin Collins, of East Finchley and Clerkenwell, bookbinder, left £630 each to the Great Eastern Hospital for Children, the London Hospital, and the Tottenham Hospital.

### THE GRADUATES' MEDICAL SCHOOLS.

DIARY FOR THE WEEK, MAY 24 TO MAY 29.

### LONDON SCHOOL OF CLINICAL MEDICINE, Seamen's Hospital, Greenwich, S.E.

**At 3.30 p.m.**
**May 26,** Mr. Cargill, **Glaucoma; its Symptoms, Physical Signs, and Pathology.**

**At 3.15 p.m.**
**May 28,** Mr. McGavin, **Psoas Abscess; its Origin and Surgical Treatment.**

### NORTH-EAST LONDON POST-GRADUATE COLLEGE, Prince of Wales's General Hospital, Tottenham, N.

**At 4.30 p.m.**
**May 25,** Dr. B. Price, **The Various Forms of Congenital Heart Disease and their Diagnosis.**
**May 27,** Mr. H. W. Carson, **Catarrhal Deafness.**

### MEDICAL GRADUATES' COLLEGE AND POLYCLINIC, 22 Chenies Street, W.C.

**At 4 p.m.**
**May 24,** Dr. H. G. Adamson, **Skin.**
**May 25,** Dr. C. Theodore Williams, **Medical.**
**May 26,** Mr. Raymond Johnson, **Surgical.**
**May 27,** Sir Jonathan Hutchinson, **Surgical.**
**May 28,** Mr. Ernest Clarke, **Eye.**

**At 5.15 p.m.**
**May 24,** Dr. F. J. Smith, **The Treatment of Typhoid Fever.**
**May 25,** Dr. T. N. Kelynack, **Open-air Schools for Tuberculous Children.**
**May 26,** Dr. R. W. Allen, **The Pathology and Treatment of a Common Cold.**
**May 27,** Sir J. Broadbent, Bart., **Relation of Arteriosclerosis to Aneurysm and Renal Disease.**

### THE POST-GRADUATE COLLEGE, West London Hospital, Hammersmith, W.

**At 10 a.m.**
**May 24 and 27,** Surgical Registrar, **Demonstration.**
**May 28,** Medical Registrar, **Demonstration.**

**At 12 noon.**
**May 24,** Dr. Bernstein, **Pathological Demonstration.**

**At 12.15 p.m.**
**May 25 and 26,** Dr. Pritchard, **Practical Medicine.**

**At 5 p.m.**
**May 24,** Mr. Lloyd, **Anæsthetics.**
**May 25,** Dr. Saunders, **Clinical Lecture.**
**May 26,** Dr. Beddard, **Medicine—II.**
**May 27,** Mr. Dunn, **Eye Diseases.**
**May 28,** Dr. Abrahams, **Skin Diseases.**

### THE THROAT HOSPITAL, Golden Square, W.

**At 5.30 p.m.**
**May 24,** Dr. Powell, **Acute Inflammatory Affections of the Middle Ear.**
**May 27,** Dr. Powell, **Chronic Purulent Otitis Media.**

### NATIONAL HOSPITAL FOR THE PARALYSED AND EPILEPTIC, Queen Sq., Bloomsbury, W.C.

**At 3.30 p.m.**
**May 25,** Dr. Batten, **Cerebellar Disease.**
**May 28,** Dr. Turner, **Epilepsy.**

### ROYAL SOCIETY OF MEDICINE, 20 Hanover Square, W.

**At 8 p.m.**
**May 24, Odontological Section** (Hon. Secs.: H. W. Trewby, D. P. Gabell, J. Howard Mummery).
Mr. Warwick James: "A Preliminary Note on the Eruption of the Teeth."

**At 5.30 p.m.**
**May 25, Medical Section** (Hon. Secs: Herbert P. Hawkins, A. M. Gossage).
Professor R. T. Hewlett: "The Treatment of Typhoid Fever with an Anti-endotoxic Serum."
Dr. E. W. Goodall and Dr. Bruce: "The Results of Treatment of Cases of Typhoid Fever with the same Anti-endotoxic serum."

**At 4.30 p.m.**
**May 28, Section for the Study of Disease in Children** (Hon. Secs.: Hugh Lett, E. I. Spriggs, Harold J. Stiles).
Dr. Parkes Weber: "Congenital Obliteration of the Bile-ducts with Cirrhosis."
Mr. A. R. Thompson: "Congenital Dislocation of Hip."

**At 8.30 p.m.**
**Section of Anæsthetics** (Hon. Secs.: Llewelyn Powell, R. W. Collum).

